# Professional Duties of the Dentist

**Published:** 1878-07

**Authors:** C. L. Kelsey


### PROFESSIONAL DUTIES OF THE DENTIST.

BY C. L. KELSEY, D. D. S.

*Read before the Northern Ohio Dental Association.*

The professional duties of the dentist is a subject which has a very broad significance. Each session of this annual meeting might, with profit be spent in discussing the various phases it embraces and yet not exhaust the subject.

As an outline of what this subject embraces we would say that it comes within the scope of the duties we owe to our professional brethren, the duties we owe to our patients, the duties we owe to community, and the duties we owe to ourselves. Every dentist, however humble his sphere, owes a duty to the profession at large. In honor he is bound to elevate and respect his profession by manly deportment and faithful, honest woik.

One who enters the profession of dentistry from mere mercenary motives, and whose aim is to make the most for himself in the shortest time, will never be of much honor to his profession.

Society will not greatly respect and honor a profession with no higher aims among its members. The duties we owe to our professional brethren are somewhat defined in our code of ethics, but outside of this there are duties of a general character which we should not overlook. What we need, is a more fraternizing, genial disposition and spirit among ourselves. We should try by all just means to be mutual helper, one of another. We should strive to harmonize in our works in the compensation we receive for our services, and in the instruction and advice we give to our patients.

Dissension begets discord, fault-finding and quibbling beget fault-finding and quibbling, and when a profession is at variance with itself, the community loses confidence in its members, its own dignity is destroyed and it is apt to become a mere matter of trade barter and shopping. To avoid this, we must cultivate friendly relations with each other and strictly adhere to our code of ethics and to a uniformity of prices for work.

From mutual help and encouragement, our annual meetings are of great importance. I think we never attend these meetings without carrying away with us a more hopeful and helpful spirit; and it is one of the professional duties of the dentist to attend and aid at the annual gatherings of his association.

These annual meetings are helpful to us in various ways, it is true they sometimes take the self-conceit out of us and reveal to us our ignorance, and we go back to our work determined to know more and do better. The duties we owe to our patients and patrons are not less important than those we owe to our profession. Affability, kindness and firmness are qualities a dentist must possess and often exercise in his dealings with his patients. He is sometimes called upon to inflict severe pain, and he should never add to this infliction rough manners or harsh words. Sympathy and kindness are sometimes better than any anæsthetic, local or general.

A dentist may be thoroughly skilled in his profession, but unless he has excellent qualities, his road to success will not be found very easy. The future welfare of the patient should not be overlooked by the dentist. We are sometimes apt to think that our whole duty to our patients is performed when we have filled their teeth, put them in good condition, and their money in our pockets; but this is only a part of our duty towards them; there may be habits in regard to the care of the teeth which need correcting, and which, if we have the confidence of patients, can be done in such a manner as not to give offense.

There may be some constitutional tendencies which need remedial agencies, and these should not be overlooked. The dentist should insist upon seeing his patients at stated intervals in order to insure permanency in the preservation of these teeth.

Then again; there are duties we owe to society at large. The calling of a dentist is such that he has to do with all classes and conditions of people, and a thorough knowledge of human nature will be of great value to him. Society expects and demands more of a professional man than of those in the humble avocations of life. I think that community expects that a professional man shall know something, and be somebody outside of his profession; and if he enters society with refined manners and an affable disposition, they will add greatly to his influence, and to his success in business.

A high rank in moral standing, a lively interest in reformatory measures, will always tend to bring to a professional man the best patronage of the community.

As a rule society generally returns a correct verdict of a man's worth and skill, Sooner or later nearly every one will pass current for what he is actually worth.

Charlatanism, false pretensions, and assumptions may succeed for a time; but sooner or later they react with damaging effect upon the pretender. Michael Angelo once said to one of his students; "Do not be too particular about pointing out the nice features of statuary; if there is any great merit in it the people will find it out and you will be appreciated accordingly."

Every man's work and skill will speak for themselves and if there is any merit in them the people will find it out.

Finally there are duties we owe to ourselves. We ought to conduct our business, and husband our resources, so as to insure a long life of usefulness to ourselves and others. But how many there are of our profession, who are laid away just at the time they ought to begin to live and be most useful to community. The constant practice of dentistry is a great tax upon a man's energies. The dentist should confine himself to short and regular hours for professional work after which he should consider himself a free American citizen.

It is a very sad thing for a man to make himself a slave of his business or profession; his manhood and energy all taken out of him by the hydraulic pressure of excessive work.

Each dentist would like to excel in his profession but at the same time he should strive to be something of a man among men. Temperate in his habits, neat in his person, and genial in his intercourse with others.

In the practice of dentistry or in any other calling one should not do his level best at all times, but should keep some of his forces in reserve. Under the most favorable circumstances the faithful practice of dentistry is a heavy draft upon the nervous system, and the dentist owes a duty to himself by taking a portion of each day for rest or recreation, and in addi ion to this he should set apart a portion of each year for entire freedom from his professional duties.

